# 3D co-culture model of endothelial colony-forming cells (ECFCs) reverses late passage adipose-derived stem cell senescence for wound healing

**DOI:** 10.1186/s13287-020-01838-w

**Published:** 2020-08-14

**Authors:** Wansheng Hu, Shengqian Zhu, Mimi Lalrimawii Fanai, Jing Wang, Junrong Cai, Jingwei Feng

**Affiliations:** Department of Plastic and Reconstructive Surgery, Nanfang Hospital, Southern Medical University, Guangzhou, 510515 China

**Keywords:** Endothelial colony-forming cells, Adipose-derived stem cells, 3-D co-culture, Wound healing

## Abstract

**Background:**

Extensive passage of adipose-derived stem cells (ASCs) in vitro leads to loss of function. Endothelial colony-forming cells (ECFCs) can be isolated from adult peripheral blood. A 3D co-culture system may rescue in vitro ASC senescence.

**Methods:**

A 3D co-culture model was successfully established using hyaluronic acid (HA) gel and a 10:1 ratio of late-passage ASCs and ECFCs. Cell density and culture conditions were optimized. Stem cell phenotype was characterized by flow cytometry. ELISA was used to measure the trophic effect of angiogenic growth factors and compare the effects of these factors between the 3-D co-culture and single-cell culture. Therapeutic potential of ASC/ECFC 3-D co-cultures was evaluated in a mouse chronic injury model.

**Results:**

Following incubation in a HA substrate 3D co-culture system, ASC morphology, phenotype, secretory profile, and differentiation capacity were restored. The ASC/ECFC co-culture increased the secretion of cytokines, such as hepatocyte growth factor, compared with single-cell 3D culture or monolayer culture. Mice radiation-ulcer wounds treated with ASC/ECFC 3-D co-cultures (spheroids) showed epithelialization and improved healing compared with wounds treated with ASCs or ECFCs alone. Further, transplanted ASC/ECFC spheroids exhibited superior angiogenic potential due to the ability of the ASCs to transdifferentiate into pericytes.

**Conclusion:**

3D co-culture of ECFCs and ASCs in vitro restored native ASC properties by reversing cellular senescence and loss of trophic function. Transplant of ASC/ECFC 3D spheroids in vivo demonstrated pro-angiogenic capacity with improved therapeutic potential.

## Background

Chronic wounds inflict a significant burden upon the physical, psychological, and financial welfare of patients, as well as the healthcare system that supports them. Unfortunately, current treatment options are limited and ineffective. Given the high rate of depression reported in patients with chronic wounds, new treatments need to be developed for improved therapeutic outcomes.

Advances in regenerative medicine, including the development of stem cell therapy, have led to possible treatments for chronic wounds. Human adipose-derived stem cells (ASCs) are an abundant source of multipotent adult mesenchymal stem cells that are easily obtained from subcutaneous adipose tissue via liposuction. ASCs have therapeutic potential because of their ability to self-renew and differentiate into multiple cell types, including adipocytes, osteocytes, chondrocytes, hepatocytes, myocytes, neurons, and monocytes. In recent years, there has been a substantial increase in the number of clinical trials using human ASCs based on their established roles in immune modulation, trophic function, multipotency, and accessibility from patients [1]. However, a single treatment may require approximately 10–800 million cells to repair a dermal wound [2–4]. Thus, it is essential to culture and expand ASCs in vitro before they can be used therapeutically.

An established characteristic of in vitro cultured ASCs is cellular senescence, resulting in proliferative arrest and dramatic changes in cell morphology, metabolism, gene expression, and secretory phenotype [5]. Implanted senescent ASCs exhibit poor survival and growth rates, reduced differentiation capacity, poor homing, and paracrine activity. To maintain ASC activity and function in vitro, novel methods reversing cellular senescence need to be investigated further.

Several factors can influence adult stem cell survival and senescence in vitro, including chemical supplementation (endogenous or exogenous growth factors), experimental preconditioning (hypoxia, growth factors, or conditioned medium), and the culture system (adherent, 3D culture, co-culture, or substrate matrices) [6, 7]. Cell-cell interaction is important for stem cell functionality. Co-culturing systems involving multiple cell types can stimulate the release of endogenous growth factors and cytokines and promote cell-cell interaction in vitro [8]. Alternatively, 3D culture models demonstrate enhanced cell-cell interaction but develop a hypoxic core [9]. Together, these previous studies show that different culture techniques can enhance or contribute to ASC senescence in vitro.

In the stem cell biology field, the recent development of three-dimensional (3D) cell-culture techniques has generated great interest as they exhibit self-patterning that mimics the in vivo development of tissues and improve progeny differentiation and functionality. Compared with suspension cultures, 3D cell culture of stem cell spheroids improves the functionality of integrated cells when injected into local host tissues. Recent reports show that non-cross-linked hyaluronic acid (HA) can sustain ASC 3D culture in vitro by providing structural stability [6]. Further, HA-cultured ASC spheroids promote regenerative capacity, plasticity, and growth factor-secreting potential of these cells. Current protocols to generate spheroids are restricted to early passage ASCs (P1–P2), limiting the scalability of this technique. Methods to sustain ASC stemness in a HA-cultured 3D model system are required to improve their therapeutic potential.

Endothelial colony-forming cells (ECFCs) are a type of progenitor cell circulating in the blood that can differentiate into several endothelial lineage types. They are easy to obtain from peripheral blood. ECFCs are characterized by a rapid proliferation rate and high levels of telomerase activity. ECFCs are believed to play a role in vascular repair after injury [10], and their therapeutic potential for skin repair or regeneration is currently being explored as an alternate tissue engineering strategy [11]. ECFCs, with great biological function, might be good candidates to provide cell-cell interaction to stimulate ASC and restore their senescence.

Herein, we describe an effective protocol to sustain ASC stemness and prevent cellular senescence in vitro by combining non-cross-linked HA and ECFC co-culture in a 3D culture model system. To evaluate their therapeutic potential for wound repair, ECFC-ASC spheroids were transplanted into mice radiation-induced ulcers.

## Material and methods

### Ethical considerations

Patient biopsies were performed in accordance with relevant government guidelines and approved by the institutional review board of the Southern Medical University. Each patient provided informed consent.

All experimental procedures using animals were performed in accordance with relevant guidelines and approved by the animal experimental committee of the Southern Medical University.

### Isolation and culture of human mesenchymal stem cells (MSCs) and ECFCs

Primary human ASCs were isolated from white adipose tissue obtained during surgical liposuction procedures as previously described [9]. ASCs were maintained in an adherent monolayer culture on untreated tissue culture plates for up to nine passages in MSC medium (MSCGM (Lonza) supplemented with 10% FBS (Hyclone)). Human ECFCs were isolated from peripheral blood as described previously [12]. ECFCs were cultured on 1% gelatin-coated plates using ECFC medium (EGM-2 (Lonza) supplemented with hydrocortisone and 20% FBS). ECFCs were used for experiments after secondary colony formation (passage number 3–5). All cell cultures were maintained in a humidified incubator at 37 °C with 5% CO_2_, unless otherwise stated.

### 3D cultures with HA gel

**Fig. 1:**
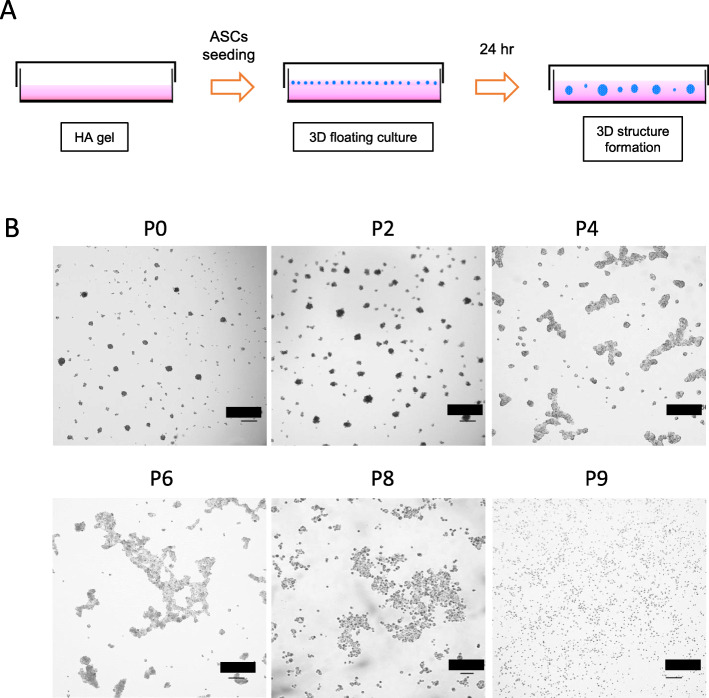
3D culture of ASCs. **a** Schematic diagram of the experimental procedure for generating the 3D culture system in HA gel. Four percent HA gel was prepared and let dissolve for hours before ASC seeding. ASC suspension was seeded to make final HA concentration of 3%. After 24 h of culture, 3D structure could form in the HA gel. **b** Morphology of ASCs grown in monolayer culture at sequential passages (P0, 2, 4, 6, 8, 9). Scale = 100 μm

Prior to experimentation, HA gel was prepared by mixing HA powder and culture medium overnight in a plastic dish (Fig. 1a). The HA powder was a pharmaceutical-grade product and non-cross-linked with an average molecular weight of 1000 kDa (Kikkoman, Tokyo, Japan). Monolayer cultures of ASCs at nine passages (p0, p2, p4, p6, p8, p9) were trypsinized, and the cells were counted and resuspended in 1 ml DMEM culture medium containing 3% (w/v) HA gel at a density of 1–6 × 10^6^/35 mm dish or 0.11–0.68 × 10^5^/cm^2^. Suspended ASCs were incubated for 24–72 h. In the presence of HA gel, 3D ASC aggregates formed in suspension.

To produce 3D co-cultured ASC/ECFC spheroids, 4 × 10^5^ ASCs (passage 8) were co-cultured in suspension with 4 × 10^4^ ECFCs in the presence of 3% HA gel for 24 h.

### Flow cytometry

For flow cytometry analysis, suspended ECFCs and ASCs were filtered, washed, and stained with the following antibodies and corresponding isotype controls: anti-CD45-Cy5 (Miltenyi Biotec, Bergisch Gladbach, Germany), anti-CD133-PE (eBioscience, Inc., CA, USA), anti-CD31-FITC (BD Biosciences), anti-CD105-FITC (BD Biosciences), rat IgG2a Kappa cy5 (BD Biosciences), and rat IgG2a Kappa Control PE (BD Biosciences). Samples and controls were analyzed with a MACSQuant Analyzer 10 (Miltenyi Biotec). Each channel was gated respective to the control samples.

### ELISA

3D cultured ASCs, ECFC, or ASC+ECFC (10% of ASCs) co-cultures were extracted from HA gels. Monolayer-cultured ASCs, ECFCs, or ASC+ECFC (10% of ASCs) were seeded at a density of 1.0 × 10^5^ cells in 6 cm^2^ plates and cultured in full growth medium for 24 h. Subsequently, the medium was exchanged for serum-free DMEM, and each dish was incubated under normoxic (6% O_2_) conditions. After 48 h, the culture media was collected and filtered through a 0.22-μm syringe filter prior to analysis. Commercially available enzyme-linked immunosorbent assay (ELISA) kits (Ray Biotech, Inc., Norcross, US) for hepatocyte growth factor (HGF), vascular endothelial growth factor (VEGF), epidermal growth factor (EGF), and platelet-derived growth factor (PDGF) were used. The absorbance was measured at 450 nm using an infinite microplate reader (Tecan Group, Männedorf, Switzerland).

### Nude mice radiation-ulcer model and treatments

Twelve-week-old male nude mice (BALB/cAJcl-FOXN1nu/nu) were purchased from Southern Medical University Experimental Animal Center (Guangzhou, China). Prior to experimentation, mice were anesthetized by isoflurane inhalation. Anesthetized mice were then placed in a lateral position and the dorsal skin pulled back to create a skin fold. The remaining body and tail were covered with a 3-mm lead shield, leaving the dorsal skinfold unprotected, which was treated with 15 Gy ionizing radiation using an MX-160Labo X-ray radiation machine (mediXtec, Japan). Experimentally treated mice were housed separately for 4 weeks. To induce a chronic injury, an 8-mm full-thickness cutaneous ulcer was created on the back of each mouse as described previously [9].

There were four groups: (a) ASC+ECFC group: irradiated mice with cutaneous ulcer treated by 3D cultured hASC (product from 4 × 10^5^ passage 8 hASCs + 4 × 10^4^ hECFC, cultured in 3% HA gel for 24 h); (b) ECFC group: monolayer cultured 4 × 10^5^ ECFCs suspended in 3% HA gel in DMEM; (c) ASC group: monolayer cultured 4 × 10^5^ hASC (passage 8) suspended in 3% HA gel in DMEM; and (d) vehicle group: 3% HA gel in DMEM. *n* = 3 mice/group and we photographed the wounds on days 0, 4, 7, 14, and 18.

### DiI labeling of ASCs

ASCs (passage 8) were cultured in suspension in a petri dish with DMEM supplemented with 10% FBS. To label, ASCs were collected and suspended with 2 μM CM-DiI (Thermo Fisher Scientific) in Hank’s balanced salt solution (HBSS) at 37 °C for 5 min, followed by incubation at 4 °C for 15 min. Labeled ASCs were then re-plated back onto the petri dish for maintenance. The staining procedure was repeated daily for 2 subsequent days until the optimal cellular fluorescence was obtained.

### Immunofluorescent analysis

3D spheroids were fixed and set in paraffin blocks as previously described. To prepare for immunolabeling, the paraffin blocks were sectioned with a cryostat, deparaffinized, and rehydrated. To retrieve antigens, sections were incubated in target-retrieval solution followed by boiling water for 20 min. Sections were permeabilized and then blocked with 1% BSA buffer. All primary and secondary antibody solutions were prepared in TBST (0.1% Tween 20, 1% BSA). Sections were incubated with guinea pig anti-Perilipin/PLIN1 (1:1000, Progen, Heidelberg, Germany) and goat anti-α smooth muscle actin (α-SMA, N-term, GeneTex, TX, USA) antibodies for 16 h at 4 °C. After a series of washes, the sections were incubated with Isolectin IB4 Alexa Fluor dye conjugates (1:1000, Invitrogen) or Hoechst 33324 (1:1000 Thermo Fisher Scientific) for 1 h. Stained sections were washed three times and then mounted. Stained sections were imaged with a fluorescent microscope. Vessel density/viable fat tissue quantification was based on α-SMA or perilipin labeling. A representative field of 500 × 500 μm was imaged for each sample (*n* = 5) in each treatment group. Fluorescent intensity was measured with ImageJ (imagej.nih.gov/ij/).

### Statistical analyses

Wound size in the irradiated mouse model was compared by the Kruskal-Wallis test. The viability assay and fluorescent intensity experiments were analyzed by one-way ANOVA and Tukey’s HSD post hoc. All statistical analyses were performed with the statistical program available at https://astatsa.com/. All statistical results were represented by the mean ± standard error. *p* values < 0.05 were considered as statistically significant.

## Results

### Late-passage ASCs fail to form 3D spheroids

In the presence of HA gel, ASC cultures at early passage (0–2) were observed to produce uniform-sized spheroids, while cultures at 4–6 passages produced irregularly shaped structures filled with cell aggregates. (Fig. 1b). When cultured in monolayers, passage 8 ASCs were either spindle-shaped or large, flat, and irregularly shaped, which are characteristics of cellular senescence. Further, late-passage ASCs were unable to form 3D spheroids in the presence of HA gel.

### ECFC characterization

**Fig. 2:**
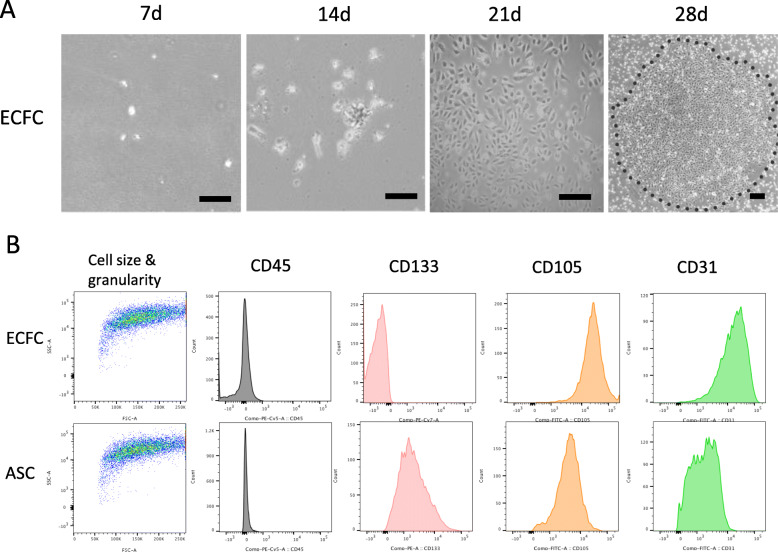
ECFC culture and characterization. **a** ECFC primary cultures imaged at day 7, 14, 21, and 28 post-isolation. Dotted line delineates the ECFC colony boundary. **b** FACS analysis of ASCs and ECFCs quantified for cell-surface markers CD45, CD133, CD105, and CD31. Scale bar = 50 μm

ECFCs were identified by positive expression of characteristic markers including CD31, VEGFR2, eNOS, and CD105, and negative expression of CD133 and CD45 [13]. ECFCs are named for their ability to form colonies of cells. Primary ECFCs were cultured for 28 days until colonies were observed and could be selected for further expansion (Fig. 2a). Flow cytometry analysis revealed that ECFCs and ASCs had similar size and granularity. Compared with primary ASCs, ECFCs are positive for the mesenchymal marker CD105 and endothelial marker CD31, and negative for the hematopoietic marker CD45 and stem cell marker CD133 (Fig. 2b). Thus, it was confirmed that the isolated primary cells were ECFCs.

### 3D co-culture of late-passage ASC and ECFCs

**Fig. 3:**
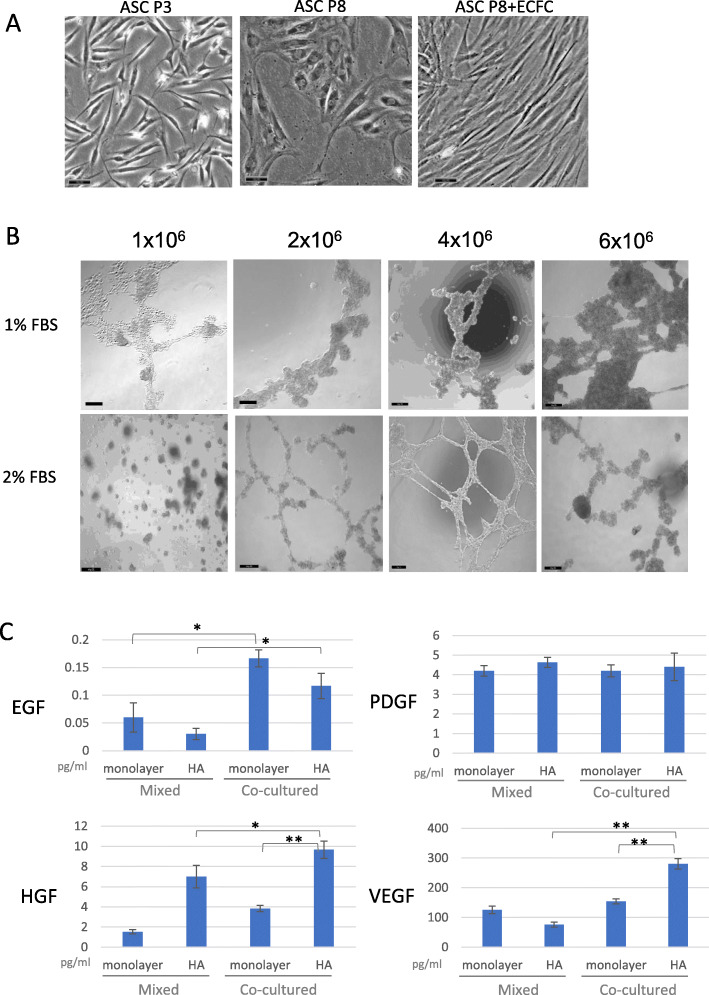
ASC/ECFC co-culture in 2D and 3D. **a** ASC morphology in monolayer culture after co-culture with ECFCs. Scale bar = 10 μm. **b** Optimization of 3D co-culture model. Scale bar = 50 μm. **c** ELISA quantification of secreted growth factors. *p < 0.05, **p < 0.01

To generate a co-culture, ECFCs were seeded on a monolayer culture of ASCs (passage 6). In the presence of ECFCs, passage 8 ASCs were observed to maintain their spindle-shaped morphology (Fig. 3a). To determine the most efficient protocol for generation of 3D co-cultures, the seeding density of ASCs was optimized (1 × 10^6^–6 × 10^6^) together with serum supplementation (1 or 2.5% FBS). Medium containing 2% FBS provided improved cell mobility compared with 1% FBS, as indicated by the large cell aggregates. Further, an ASC seeding density of 4 × 10^6^ in cultures supplemented with 2% FBS promoted the formation of tubular-like structures in the HA gel (Fig. 3b). We speculate that this structure may represent the neovascular properties of endothelial cells.

### Trophic functionality of 3D co-culture spheroids

The secretory functions of 3D co-cultures were assessed by ELISA. Immunological analysis revealed that secretions of VEGF, HGF, and EGF were generally higher under co-culture conditions than when mixed conditioned media were employed. The ASC+ECFC co-culture promoted the secretion of HGF/EGF when compared with the conditioned medium mixture from both 2D and 3D monoculture conditions. PDGF secretion did not differ between the groups. The 3D co-culture model significantly enhanced the secretion of VEGF and HGF compared with the other experimental groups (Fig. 3c).

### Viability and characterization of 3D co-cultured cells

**Fig. 4:**
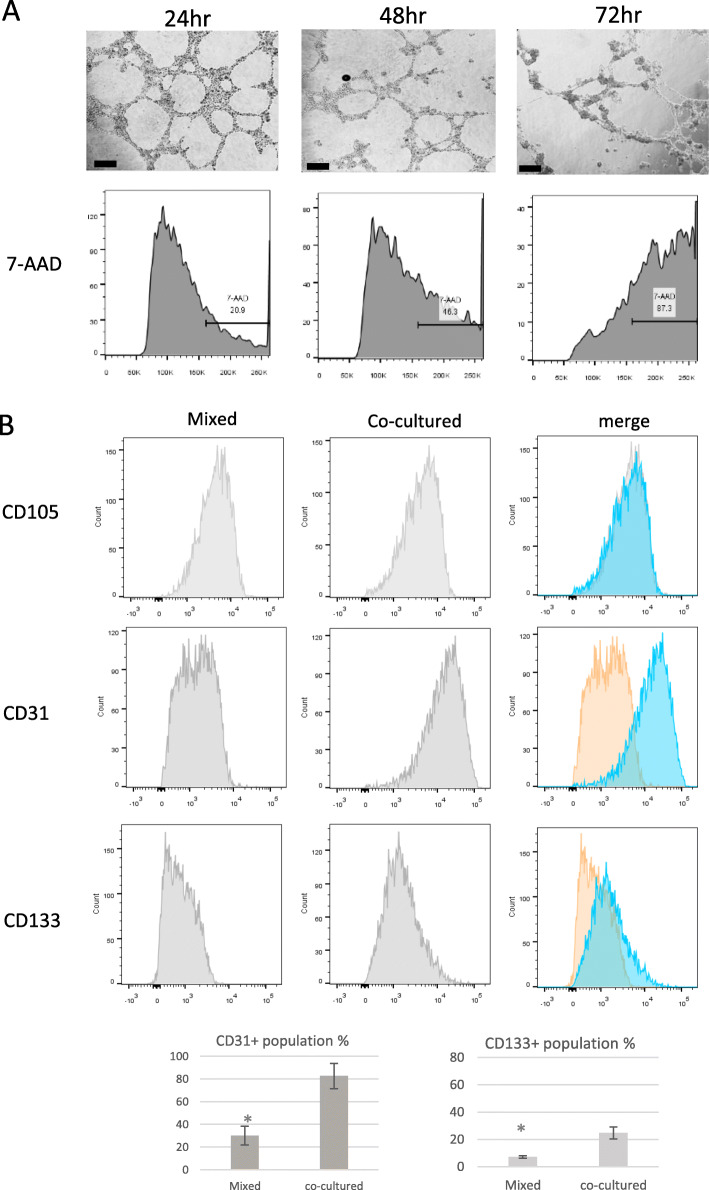
Cell viability and characterization of 3D cultured cells. **a** Apoptosis assay. Flow cytometry analysis for 7-AAS staining of 3D co-cultured cells. **b** Cell characterization of 3D co-cultures

Cell viability of the 3D co-culture spheroid was assessed by flow cytometry using the 7-AAD stain. Over time, cell death increased after 24 h (22.1 ± 4.3%), 48 h (45.8 ± 7.1%), and 72 h (90.5 ± 9.0%) incubation (Fig. 4a). Twenty-four hours incubation was therefore considered as the most favorable culture period for achieving reduced cell death. 3D co-culture spheroids incubated in HA gel for 24 h were enzymatically suspended into single cells for characterization by flow cytometry analysis. Like the mixed cultures, the 3D co-culture maintained the expression of the mesenchymal marker CD105. In comparison, there was an increase in the CD31 (approx. 2.8-fold and CD133 (approx. 3-fold) positive populations when cells were co-cultured in 3D (Fig. 4b)). These results indicated that the ASCs were undergoing transdifferentiation.

### Chronic injury assessment of radiation-ulcer mice

**Fig. 5:**
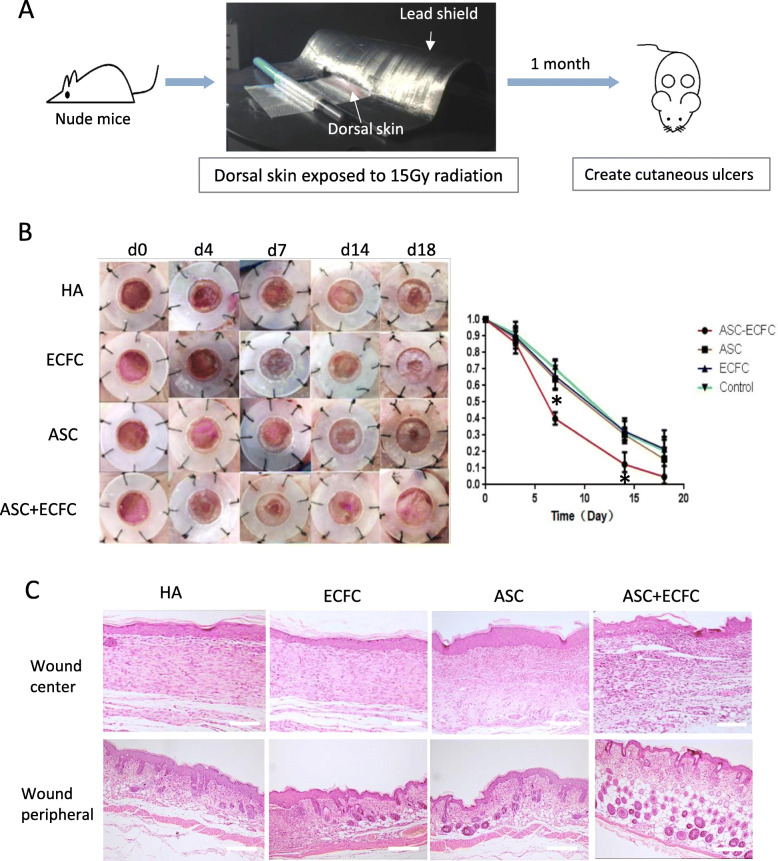
Nude mice radiation ulcer model. **a** Schematic diagram of the radiation ulcer model. **b** Images representing wound healing. Left panel shows macroscopic views of the ulcer. Right panel is the wound healing curve. The ASC/ECFC group demonstrated the fastest re-epithelization. **c** H&E staining of the wound center and periphery. Scale = 100 μm

Overall, ulcers created in the irradiated mouse tissues showed impaired healing compared with those in the normal tissue. Eighteen days post-injury, the vehicle-treated (HA) ulcers in the irradiated tissue remained unhealed (Fig. 5b). Seven days post-injury, ASC/ECFC-treated ulcers showed improved re-epithelialization and wound healing (wound area 40.1 ± 3.0%) compared with ulcers treated with only ASCs (63.3 ± 6.7%) or ECFCs (65.8 ± 7.2%). The late-passage ASC-treated ulcers healed similarly to vehicle-treated ulcers (76.16 ± 5.2%) (*p* > 0.05). Fourteen days post-injury, the ASC/ECFC-treated ulcers had healed more (77% wound closure) than those in mice in the other treatment groups, which showed significantly impaired healing.

Histological evaluation of the ulcer wound demonstrated the formation of scar tissue in the central area (Fig. 5c). Despite normal dermal thickness, ASC/ECFC-treated mice had a thicker subcutaneous adipose layer in the wound marginal area. ASC/ECFC-treated mice exhibited more collagen deposition in the wound center.

**Fig. 6:**
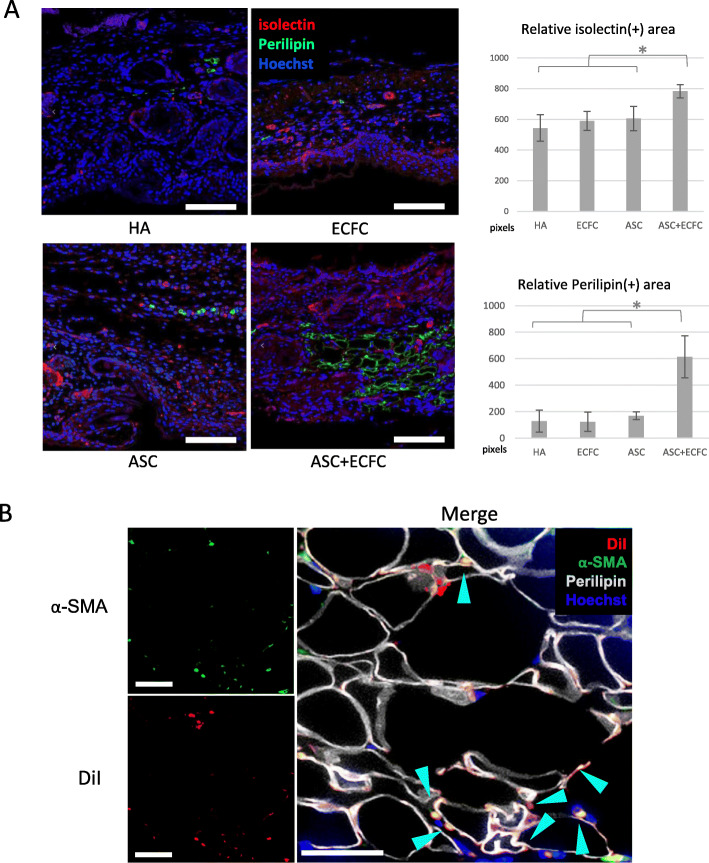
Evaluation of vascularization. **a** Immunofluorescence of isolectin (vasculature) and perilipin (adipocytes) of day 14 samples and respective semi-quantification. p* < 0.05. Scale bar = 100 μm. **b** Immunofluorescence staining of the regenerated dermis. Double positive cells are stained orange (cyan arrow heads). Scale = 20 μm

Microscopic differences in the ulcer vasculature were noted between ASC/ECFC-treated ulcers and other treatment groups. To investigate further, vascularization of the wound tissue was examined by immunocytochemistry of α-SMA-stained capillaries (Fig. 6a). ASC/ECFC-treated ulcers had approximately 30% more α-SMA-positive cells than the other three treatment groups. Adipogenesis was evaluated by quantification of perilipin staining. There was a 4-fold increase in the adipose content of the ASC/ECFC ulcers compared with the other groups. However, ulcers treated with only passage 8 ASCs or ECFCs alone did not differ from the vehicle control.

To trace transplanted ASCs, DiI labeling was performed. Eighteen days post-transplantation, cells in the deep subcutaneous layer of ASC/ECFC ulcers were arranged in circular patterns, a proportion of which was positive for both α-SMA and DiI (Fig. 6b). Double-positive cells indicated that the engrafted ASCs were developing into blood vessels in the host tissue.

## Discussion

Co-cultured cellular models combining the endothelial and mesenchymal lineage hold great potential for tissue therapy as they enable development of pre-vascularized tissue constructs. In this study, our aim was to rescue ASC senescence and loss of function that is a consequence of extended passage in vitro. In vitro co-culture of late-passage ASCs with a 1:10 ratio of ECFCs and HA gel was shown to restore native ASC function, morphology, and phenotype, by reversing cellular senescence. When transplanted into mice with a chronic injury, the 3D co-culture was demonstrated to have pro-angiogenic capacity and promote healing.

In this study, HA was used to construct a 3D structure of cell spheroids. 3D spheroids have been reported to produce growth factors that promote cell survival and growth that improves upon adherent culture. HA is a naturally occurring molecule in the extracellular matrix of human tissues. It is a glycosaminoglycan disaccharide composed of alternately repeating units of D-glucuronic acid and *N*-acetylglucosamine. HA is a degradable material widely used for both pharmaceutical and cosmetic purposes; therefore, it is a therapeutically safe alternative. In this study, non-cross-linked HA powder was combined with culture medium to generate a matrix solution to promote 3D formation. HA-formed 3 spheroids were easily dissociated without enzyme digestion. 3D ASC spheroid formation has been reported to promote pluripotency as demonstrated by upregulation of stem cell markers [6, 9]. Together, these results demonstrate that HA is a practical material for 3D cell culture with therapeutic potential for tissue engineering.

From a translational standpoint, this study demonstrates that ECFC co-culture provides the means to improve the efficiency of ASC transplantation. Numerous clinical trials have tested the therapeutic value of MSC/ASC-based treatments for ischemic disease. However, due to poor engraftment capabilities of MSCs, these therapies remain ineffective [14, 15]. Emerging evidence indicates that the endothelial lineage can regulate homeostatic and regenerative processes via paracrine production of trophic factors, including pancreatic differentiation, liver organogenesis, hematopoietic stem cell progenitor proliferation, neurogenesis, and osteogenesis [16–20]. Cell-to-cell signaling between mesenchymal stem and endothelial cell (MSC-EC) not only promotes cytokine secretion, but also facilitates engraftment and regenerative capacity [21, 22]. Further research is necessary to explore the mechanisms behind the paracrine effect of ECFCs on ASC senescence. This study highlights the importance of maintaining MSC-EC communication for preventing ASC senescence in vitro.

Products with regenerative function were shown to improve their efficacy and stability by combining HA. Previous studies have shown that HA can carry platelet-rich plasma (PRP) and enhance its efficacy by stabilizing the platelets and prolonging its cytokine release [23–25]. Stromal vascular fraction (SVF) cells are the other cell type that is frequently used for regenerative purpose. They can be easily isolated from human adipose tissue and contain a variety of functional cells including ASCs [26, 27]. SVF cells were proved to be efficient and safe to use SVF cells to improve fat graft retention and other regenerative purposes [28–31]. ASCs were obtained from SVF cells and proved to be the key functional cell population in SVF cells [32–34]. Combination of SVF or ASCs with HA was proved to have great potential for the development of new therapeutic approaches to improve the treatment safety and efficacy [35–37].

Our results indicate that 3D ASC/ECFC spheroids have neovascular potential, as demonstrated by the presence of perivascular cells that promote angiogenesis in situ. In vivo, transplant of the 3DASC/ECFC spheroid demonstrated angiogenesis, as indicated by the presence of α*-*SMA-positive cells in the capillary walls and differentiated pericytes. Under normal conditions, this property can only be achieved with early passage ASCs [6, 9]. Thus, co-culture with HA enables late-passage ASCs to undergo transdifferentiation into pericytes for neovascularization.

## Conclusion

Our study demonstrates that ECFCs can restore stem cell properties of late-passage ASCs in vitro*.* Optimization of a 3D co-culture model of ASC/ECFCs by the addition of HA gel promoted plasticity and growth factor-secreting potential of these cells. Further, 3D co-culture spheroids improved wound healing in a mouse model of chronic injury by promoting engraftment efficiency and angiogenesis. Together, 3D co-cultures of ASC/ECFCs may provide an alternative for tissue engineering purposes.

## Data Availability

All data generated or analyzed in this study are included in this article.

## Abbreviations

ASCs: Adipose-derived stem cells
ECFCs: Endothelial colony-forming cells
HA: Hyaluronic acid
3D: Three-dimensional
MSCs: Mesenchymal stem cells
ELISA: Enzyme-linked immunosorbent assay
HGF: Hepatocyte growth factor
VEGF: Vascular endothelial growth factor
EGF: Epidermal growth factor
PDGF: Platelet-derived growth factor

## References

[1] Cherubino M, Marra KG (2009). Adipose-derived stem cells for soft tissue reconstruction. Regen Med.

[2] Bura A (2014). Phase I trial: the use of autologous cultured adipose-derived stroma/stem cells to treat patients with non-revascularizable critical limb ischemia. Cytotherapy.

[3] Darinskas, Adas, and Mindaugas Paskevicius."Stromal vascular fraction cells for the treatment of critical limb ischemia: a pilot study." J Transl Med 15.1(2017). 10.1186/s12967-017-1243-3 PMC5477131 28629476

[4] Han SK, Kim HR, Kim WK (2010). The treatment of diabetic foot ulcers with uncultured, processed lipoaspirate cells: a pilot study. Wound Repair Regen.

[5] Legzdina D (2016). Characterization of senescence of culture-expanded human adipose-derived mesenchymal stem cells. Int J Stem Cells.

[6] Mineda K (2015). Therapeutic potential of human adipose-derived stem/stromal cell micro-spheroids prepared by 3D culture in non-cross-linked hyaluronic acid gel. Stem Cells Transl Med.

[7] Khatiwala R, Cai C (2016). Strategies to enhance the effectiveness of adult stem cell therapy for ischemic heart diseases affecting the elderly patients. Stem Cell Rev.

[8] Mckee, Christina, and G. R. Chaudhry. "Advances and challenges in stem cell culture." *Colloids and surfaces B: Biointerfaces* 159(2017). 10.1016/j.colsurfb.2017.07.051 28780462

[9] Feng, Jingwei, et al. "An injectable non-cross-linked hyaluronic-acid gel containing therapeutic spheroids of human adipose-derived stem cells." Scientific Reports 7.1(2017):1548. 10.1038/s41598-017-01528-3 PMC5431556 28484208

[10] Ingram, and A. D. . "Identification of a novel hierarchy of endothelial progenitor cells using human peripheral and umbilical cord blood." Blood 104.9(2004):2752–2760. 10.1182/blood-2004-04-1396 15226175

[11] Hendrickx B (2010). Integration of blood outgrowth endothelial cells in dermal fibroblast sheets promotes full thickness wound healing. Stem Cells.

[12] Juan M Melero-Martin, Zia A Khan, Arnaud Picard, et al. In vivo vasculogenic potential of human blood-derived endothelial progenitor cells. Blood, 2007, 109(11):4761–4768. 10.1182/blood-2006-12-062471 17327403

[13] Hur J (2004). Characterization of two types of endothelial progenitor cells and their different contributions to neovasculogenesis. Arterioscler Thromb Vasc Biol.

[14] Jacques G, Luc S (2018). Mesenchymal stromal cells: clinical challenges and therapeutic opportunities. Cell Stem Cell.

[15] Gerhard, et al. "Concise review: a comprehensive analysis of reported adverse events in patients receiving unproven stem cell-based interventions." Stem Cells Transl Med. 2018;7(9):676–85. 10.1002/sctm.17-0282 PMC6127222 30063299

[16] Lammert E, Cleaver O, Melton D (2001). Induction of pancreatic differentiation by signals from blood vessels. Science.

[17] Matsumoto K, Yoshitomi H, Rossant J, Zaret KS (2001). Liver organogenesis promoted by endothelial cells prior to vascular function. Science.

[18] Butler JM (2010). Endothelial cells are essential for the self-renewal and repopulation of Notch-dependent hematopoietic stem cells. Cell Stem Cell.

[19] Shen Q (2004). Endothelial cells stimulate self-renewal and expand neurogenesis of neural stem cells. Science.

[20] Kaigler D (2005). Endothelial cell modulation of bone marrow stromal cell osteogenic potential. FASEB J.

[21] Lin RZ (2014). Human endothelial colony-forming cells serve as trophic mediators for mesenchymal stem cell engraftment via paracrine signaling. Proc Natl Acad Sci.

[22] Thin Luu N (2013). Crosstalk between mesenchymal stem cells and endothelial cells leads to downregulation of cytokine-induced leukocyte recruitment. Stem Cells.

[23] Nicoli F, Balzani A, Lazzeri D (2015). Severe hidradenitis suppurativa treatment using platelet-rich plasma gel and Hyalomatrix. Int Wound J.

[24] Gentile P, Bottini DJ, Spallone D (2010). Application of platelet-rich plasma in maxillofacial surgery: clinical evaluation. J Craniofac Surg.

[25] Cervelli V, Lucarini L, Spallone D et al. "Use of platelet-rich plasma and hyaluronic acid in the loss of substance with bone exposure". Adv Skin Wound Care. 2011;24(4):176–181. 10.1097/01.ASW.0000396302.05959.d3 21422842

[26] Gentile P, Scioli MG, Cervelli V et al. "Autologous micrografts from scalp tissue: trichoscopic and long-term clinical evaluation in male and female androgenetic alopecia". Biomed Res Int. 2020;2020:7397162. 10.1155/2020/7397162 PMC7007958 32071919

[27] Gentile P. "Autologous cellular method using micrografts of human adipose tissue derived follicle stem cells in androgenic alopecia". Int J Mol Sci. 2019;20(14). 10.3390/ijms20143446 PMC6678214 31337037

[28] Gentile P, Casella D, Palma E et al. "Engineered fat graft enhanced with adipose-derived stromal vascular fraction cells for regenerative medicine: clinical, histological and instrumental evaluation in breast reconstruction". J Clin Med. 2019;8(4). 10.3390/jcm8040504 PMC6518258 31013744

[29] Gentile P, Calabrese C, De Angelis B et al. "Impact of the different preparation methods to obtain human adipose-derived stromal vascular fraction cells (AD-SVFs) and human adipose-derived mesenchymal stem cells (AD-MSCs): enzymatic digestion versus mechanical centrifugation". Int J Mol Sci. 2019;20(21). 10.3390/ijms20215471 PMC6862236 31684107

[30] Gentile P, Scioli MG, Bielli A (2017). Comparing different nanofat procedures on scars: role of the stromal vascular fraction and its clinical implications. Regen Med.

[31] Gentile P, Scioli MG, Orlandi A et al. "Breast reconstruction with enhanced stromal vascular fraction fat grafting: what is the best method?". Plast Reconstr Surg Glob Open. 2015;3(6):e406. 10.1097/GOX.0000000000000285 PMC4494476 26180707

[32] Gentile P, Sterodimas A. "Adipose-derived stromal stem cells (ASCs) as a new regenerative immediate therapy combating coronavirus (COVID-19)-induced pneumonia". Expert Opin Biol Ther. 2020;20(7):711–6. 10.1080/14712598.2020.1761322 PMC7196919 32329380

[33] Gentile P, Kothari A, Casella D et al. "Fat graft enhanced with adipose-derived stem cells in aesthetic breast augmentation: clinical, histological, and instrumental evaluation". Aesthet Surg J. 2019.sjz292. 10.1093/asj/sjz292 31637416

[34] Gentile P, Piccinno MS, Calabrese C. "Characteristics and potentiality of human adipose-derived stem cells (hASCs) obtained from enzymatic digestion of fat graft". Cells. 2019;8(3):282. 10.3390/cells8030282 PMC6469026 30934588

[35] Gentile P, De Angelis B, Pasin M et al. "Adipose-derived stromal vascular fraction cells and platelet-rich plasma: basic and clinical evaluation for cell-based therapies in patients with scars on the face". J Craniofac Surg 2014, 25(1):267–272. 10.1097/01.scs.0000436746.21031.ba 24406591

[36] Scioli MG, Bielli A, Gentile P et al. "Combined treatment with platelet-rich plasma and insulin favours chondrogenic and osteogenic differentiation of human adipose-derived stem cells in three-dimensional collagen scaffolds". J Tissue Eng Regen Med. 2017;11(8):2398–2410. 10.1002/term.2139 27074878

[37] Gentile P, Garcovich S. "Concise review: adipose-derived stem cells (ASCs) and adipocyte-secreted exosomal microRNA (A-SE-miR) modulate cancer growth and promote wound repair". J Clin Med. 2019;8(6):855. 10.3390/jcm8060855 PMC6616456 31208047

